# Study protocol for the randomised controlled trial: Antiglucocorticoid augmentation of anti-Depressants in Depression (The ADD Study)

**DOI:** 10.1186/1471-244X-13-205

**Published:** 2013-08-03

**Authors:** R Hamish McAllister-Williams, Eleanor Smith, Ian M Anderson, Jane Barnes, Peter Gallagher, Heinz CR Grunze, Peter M Haddad, Allan O House, Tom Hughes, Adrian J Lloyd, Elaine MM McColl, Simon HS Pearce, Najma Siddiqi, Baxi Sinha, Chris Speed, I Nick Steen, June Wainright, Stuart Watson, Fiona H Winter, I Nicol Ferrier

**Affiliations:** 1 Institute of Neuroscience, Newcastle University, Newcastle upon Tyne, UK; 2 Northumberland Tyne and Wear NHS Foundation Trust, Newcastle upon Tyne, UK; 3 Greater Manchester West Mental Health NHS Foundation Trust and Neuroscience and Psychiatry Unit, Manchester University, Manchester, UK; 4 Newcastle Clinical Trials Unit, Institute of Health and Society, Newcastle University, Newcastle upon Tyne, UK; 5 Institute of Health Sciences, University of Leeds, Leeds, UK; 6 Leeds and York Partnership NHS Foundation Trust, Leeds, UK; 7 Institute of Genetic Medicine, Newcastle University, Newcastle upon Tyne, UK; 8 Bradford District NHS Care Trust, Bradford, UK; 9 Tees, Esk and Wear Valleys NHS Foundation Trust, Edward Pease Way, Darlington, County Durham, UK; 10 Mental Health Research Network, North East Hub service user and carer group, Newcastle, UK; 11 Academic Psychiatry, Wolfson Research Centre, Campus for Ageing and Vitality, Newcastle upon Tyne, NE4 5PL, UK

**Keywords:** Antidepressive agents, Cortisol, Depressive disorder, Metyrapone, Antiglucocorticoid treatment, Treatment refractory depression

## Abstract

**Background:**

Some patients with depression do not respond to first and second line conventional antidepressants and are therefore characterised as suffering from treatment refractory depression (TRD). On-going psychosocial stress and dysfunction of the hypothalamic-pituitary-adrenal axis are both associated with an attenuated clinical response to antidepressants. Preclinical data shows that co-administration of corticosteroids leads to a reduction in the ability of selective serotonin reuptake inhibitors to increase forebrain 5-hydroxytryptamine, while co-administration of antiglucocorticoids has the opposite effect. A Cochrane review suggests that antiglucocorticoid augmentation of antidepressants may be effective in treating TRD and includes a pilot study of the cortisol synthesis inhibitor, metyrapone. The Antiglucocorticoid augmentation of anti-Depressants in Depression (The ADD Study) is a multicentre randomised placebo controlled trial of metyrapone augmentation of serotonergic antidepressants in a large population of patients with TRD in the UK National Health Service.

**Methods/design:**

Patients with moderate to severe treatment refractory Major Depression aged 18 to 65 will be randomised to metyrapone 500 mg twice daily or placebo for three weeks, in addition to on-going conventional serotonergic antidepressants. The primary outcome will be improvement in Montgomery-Åsberg Depression Rating Scale score five weeks after randomisation (i.e. two weeks after trial medication discontinuation). Secondary outcomes will include the degree of persistence of treatment effect for up to 6 months, improvements in quality of life and also safety and tolerability of metyrapone. The ADD Study will also include a range of sub-studies investigating the potential mechanism of action of metyrapone.

**Discussion:**

Strengths of the ADD study include broad inclusion criteria meaning that the sample will be representative of patients with TRD treated within the UK National Health Service, longer follow up, which to our knowledge is longer than any previous study of antiglucocorticoid treatments in depression, and the range of mechanistic investigations being carried out. The data set acquired will be a rich resource for a range of research questions relating to both refractory depression and the use of antiglucocorticoid treatments.

**Trial registration:**

Current Controlled Trials: ISRCTN45338259; EudraCT Number: 2009-015165-31.

## Background

Depression is a common disorder, affecting some 10% of the population [1]. It is rated by the Disease Control Priorities Project as one of the leading medical contributors to the global burden of disease [2]. It can become long-lasting and may recur frequently. Depression has a large negative impact on the quality of life of service users and their carers. It is associated with high morbidity (depression has been identified as one of the leading causes of work days lost and working-age adults receiving disability payments in the UK) [3] and mortality, through suicide and increased deaths from cardiovascular disease [4]. Clinical guidelines recommend the use of antidepressant medication for the treatment of a moderate to severe depressive episode [1,5]. Antidepressant drugs have established efficacy versus placebo in clinical trials, however, in naturalistic settings many patients have unsatisfactory outcomes. The large pragmatic STAR*D study conducted in the USA showed that even with protocol driven treatment, first line therapy with a selective serotonin reuptake inhibitor (SSRI: citalopram) in over 2500 patients was associated with remission in only 28%, and response (defined as a 50% decrease in symptom scores) in less than 50% of patients [6]. Further, second line treatment in the STAR*D study with an alternative antidepressant was associated with an even lower remission rate of 20-25% [7]. Much of the burden is consequent upon this treatment refractory depression (TRD).

Clinical response to antidepressant treatment may be influenced by a number of factors, but an inadequate neurochemical response is one likely mechanism of non-response. The majority of antidepressants, on chronic administration, elevate forebrain levels of 5-hydroxytryptamine (serotonin, 5-HT). The clinical importance of this is supported by the fact that acute depletion of tryptophan (the precursor of 5-HT) can lead to the rapid return of depressive symptoms in patients treated with antidepressants [8,9]. There is a long-held notion that life events and lack of social support predict worse treatment outcomes in patients with depression [10]. The mechanism of this may relate to the hypothalamic-pituitary-adrenal (HPA) axis. It is well established from animal and human studies that glucocorticoids influence multiple aspects of 5-HT neurotransmission (including the sensitivity of 5-HT_1A_ autoreceptors and postsynaptic 5-HT receptor efficacy [11-14] that are postulated to be central to the antidepressant mechanism of action [15]). Furthermore, dysregulation of the HPA axis can reduce the effects of antidepressants in the frontal cortex. Implantation of corticosteroid releasing pellets in rodents to induce an HPA axis dysregulation has been shown to attenuate the ability of antidepressants to elevate forebrain 5-HT levels [16]. Interestingly, there is also increasing evidence that antiglucocorticoid treatments have the opposite effect by enhancing the forebrain 5-HT response to antidepressants [17]. This suggests that reducing normal physiological levels of glucocorticoid receptor activation can increase the elevation of 5-HT in response to SSRIs. These data suggest a potential mechanism by which antiglucocorticoid strategies can enhance the clinical effectiveness of antidepressants in clinical practice.

HPA axis abnormalities are often demonstrated in patients with mood disorders. HPA axis dysregulation in depression is often characterised, particularly in those with melancholic symptoms, by a flattened cortisol diurnal rhythm with elevated trough levels of cortisol [18] and by attenuated negative feedback effects of corticosteroids on adrenocorticotrophin hormone (ACTH) and cortisol release [19]. There is increasing evidence that shows such dysregulation is associated with poor prognosis, including non-response to antidepressants and future relapse [20-26]. Further, a Cochrane review demonstrated the efficacy of antiglucocorticoid augmentation of antidepressants in patients with depression with the largest effect size seen with metyrapone [27], a cortisol synthesis inhibitor which crosses the blood–brain barrier. There have been several open or open-label, including randomised, studies of metyrapone augmentation of antidepressants, showing efficacy in treatment-resistant depression [28-32]. In addition a successful proof-of-concept double blind, placebo controlled randomised study has been conducted by Jahn and colleagues [33] in a centre in Germany, with 63 depressed inpatients. Patients were all on a serotonergic antidepressant and were randomised to add-on treatment with metyrapone (1 g/day for 3 weeks) or placebo. The primary outcome measure was the percentage of responders (defined by a set improvement on the Hamilton depression rating scale) 5 weeks after randomisation (i.e. 2 weeks after cessation of metyrapone or placebo augmentation). Patients receiving metyrapone were significantly more likely to respond than those receiving placebo, with an effect size of d = 0.63. Kaplan-Meier analysis revealed a faster response with metyrapone, which was well tolerated and without serious side-effects.

### Aims

The primary aim of the “**A**ntiglucocorticoid augmentation of anti-**D**epressants in **D**epression” (**ADD**) study is to examine the efficacy and safety of metyrapone augmentation of serotonergic antidepressants in a randomised controlled trial (RCT) in patients with Major Depression who have not responded to at least two courses of antidepressants in their current episode. The study extends research in this area by exploring the translatability of the proof of concept study described above [33] to an outpatient, primary and secondary care, UK National Health Service (NHS) population. To date, all published studies of the use of antiglucocorticoids in patients with TRD have used short treatment periods of 1-3 weeks [27], which can appear counter-intuitive in such a potentially chronic condition. However, evidence suggests that the clinical effects of antiglucocorticoids on HPA axis function persist after their prescription has ceased [34,35]. The persistence of effects on depressive symptoms and quality of life will therefore be examined in the ADD study for around 6 months after stopping metyrapone treatment compared with the 2 week follow-up period of Jahn and colleagues [33].

The exact mechanism by which metyrapone may enhance antidepressant efficacy is unknown. A secondary aim of the ADD study is to explore the impact of metyrapone on HPA axis function and the hypothesis that metyrapone leads to altered neural responsiveness to glucocorticoids with an increase in the frontocortical 5-HT response to antidepressants. In addition, a number of studies in sub-samples, drawn from the main RCT population, will also be undertaken using electroencephalographic (EEG) and functional magnetic resonance imaging (fMRI) techniques, assessment of neuropsychological function, and genetic variability to address this mechanistic aim. Only the elements of the study relating to the full RCT sample (efficacy and safety measures and HPA axis function) will be described in detail here. The sub-studies are, however, detailed in Table 1. The description of the protocol below is consistent with the SPIRIT (Standard Protocol Items: Recommendations for Interventional Trials) 2013 recommendations (see http://www.spirit-statement.org/spirit-statement/).

**Table 1:** Sub-studies: data collected and rationale

**Sub- study**	**Data collected**	**Rationale**	**Participants**
Neuropsychological function	CANTAB spatial working memory (SWM) task [36].	The neuropsychological tasks have chosen on the basis of their known sensitivity to cortisol and/or 5-HT manipulations. For example SWM has been shown to be sensitive to corticosteroid manipulations in healthy controls and patients [37-39]. The FEERT, ECMT and affective go/no-go tasks, on the other hand have strong evidence for sensitivity to 5-HT manipulations [40-42], while episodic verbal memory, as tested with the VTL) is sensitive to both corticosteroid and 5-HT manipulations [43-45]. Changes in performance of the tasks following metyrapone treatment will help identify whether any improvements with the treatment may be associated with changes in HPA axis function and/or 5-HT neurotransmission.	80 patients, equal numbers from each treatment arm. 50 patients recruited in Newcastle and 30 in Manchester.
*Patients – week 0 and 5*	Verbal Learning Test (VTL) comprising neutral, positive and negative emotional words with immediate and delayed recall and recognition trails.		
*Healthy Controls – week 0*			
			55 healthy controls.
	Object-Location Memory (OLM) paradigm [46].		
	Digit Span – forward and backward.		
	Attentional Network Test (ANT) [47].		
	Facial Emotional Expression Recognition Test (FEERT) [48].		
	Emotional Categorisation and Memory test (ECMT) [48].		
	Affective Go/No Go Task [49].		
EEG: Treatment response prediction	Alpha power.	This sub-study will utilise a range of EEG variables with an evidence base for predicting response to monoaminergic antidepressants [50]. The LDAEPs have been shown to differentially predict response to serotonergic versus noradrenergic antidepressants [51]. We predict that the relationship between clinical response and LDAEP measurement will be consistent with serotonergic enhancement by metyrapone.	50 patients, equal numbers from each treatment arm, recruited in Newcastle.
	Alpha hemispheric asymmetry.		
*Patients – week 0 and 5*			
	Theta power localised to anterior cingulate.		
*Healthy Controls – week 0*			25 healthy controls.
	Loudness dependency of auditory evoked potentials (LDAEPs).		
EEG: Neural correlates of emotional processing and memory	Emotional source memory task (ESMT) [52].	The ESMT task will examine the neural correlates of emotional episodic memory in patients and the effects of metyrapone treatment. This will supplement the information provided in the VTL in the neuropsychological sub-study described above.	50 patients, equal numbers from each treatment arm, recruited in Newcastle.
	Putative EEG measure of long-term potentiation (LTP) [55].		
			25 healthy controls.
		The putative LTP measure has previously been shown to be impaired in depressed patients [53] and LTP is known to be sensitive to corticosteroids in animals [54]. We predict that treatment with metyrapone will normalise potential impairments in this measure in the patient cohort.	
*Patients – week 0 and 5*			
*Healthy Controls – week 0*			
Newcastle fMRI sub-study	Facial emotional processing task (FEPT) [56].	FEPT allows investigation of a functional cortical network involved in the processing of emotions and emotional responses. It supplements information obtained using the FEERT in the neuropsychological sub-study described above and enable localisation of any effects seen. Likewise the EEMET will supplement the information provided in the VTL.	30 patients from NE Hub - equal number from each treatment arm.
*Patients – week 0 and 5*			
	Emotional episodic memory encoding task (EEMET) [57].		
*Healthy Controls – week 0*			15 healthy controls matched for age, handedness and IQ.
Manchester fMRI sub-study	Neural correlates of episodic and working memory.	The episodic memory task will involve encoding and recall of neutral and emotionally valenced pictures, while the working memory task will utilise the N-back task. This will further supplement the data obtained in the neuropsychological sub-study.	30 patients from NW Hub - equal number from each treatment arm.
*Patients – week 0 and 5*			
	Hydrocortisone pharmacoMRI [58].		
			30 healthy matched controls.
		For the pharmacoMRI, at week 0 patients receiving metyrapone will receive a 100 mg hydrocortisone bolus and those receiving placebo saline. Healthy controls will be randomised to hydrocortisone or saline. At week 5 all patients will receive hydrocortisone. We predict that blunted acute hippocampal responses to hydrocortisone pre-treatment will normalise after metyrapone, but not placebo, treatment.	
*Healthy Controls – week 0*			
Genotyping		We will look for an association between treatment outcome, its predictors and genetic polymorphisms and also for genetic influences on brain information processing (for example brain derived neurotropic factor (BDNF) and corticotrophin releasing factor (CRF) variants and hippocampal response to hydrocortisone in Manchester/amygdala response to emotional faces in Newcastle). We will also investigate the DNA samples for rare genetic variants (e.g. copy number variations). We will determine our candidate gene list based on previous candidate gene association studies and recent novel findings from genome-wide association studies with major depressive disorder and by considering relevant genes for the function of the HPA axis.	All patients and healthy controls approached for specific consent to provide a DNA sample.
*Patients and Controls*			

Note, all the patients will be recruited from the main ADD study RCT with additional inclusion criteria of being aged 18-60. To assist in interpretation of the data an additional cohort of healthy controls will be recruited who are currently psychiatrically well, confirmed through SCID interview and HDRS17 < 5 and being on no current psychotropic medication. Exclusion will also include presence of a past history of psychiatric illness or a history of psychiatric illness in a first degree relative. The controls will be matched with the patients for age (+/- 5 years), gender, handedness and IQ (+/- 7 points as assessed using the National Adult Reading Test [59]). Handedness will be assessed for all subjects taking part in EEG and fMRI studies (using the Edinburgh Handedness Inventory [60]). Only right handed patients and healthy controls will be included in the fMRI sub-studies described below.

## Methods/design

### Study design

The ADD study is a multicentre parallel group, double-blind, randomised, placebo-controlled superiority trial of augmentation of serotonergic antidepressants with metyrapone in patients with moderate to severe depression who have failed to respond to adequate trials of at least two antidepressants in their current episode.

### Study population

The initial study aim was to recruit 190 patients. However due to initial slow recruitment rates after discussion with the funder, sponsor and the independent Data Monitoring and Ethics Committee, a revised target of 140 was agreed by accepting a reduction in power of the study from 90% to 80% on the primary outcome measure (see below). These patients will be recruited from primary and secondary care settings.

### Inclusion criteria

•Diagnostic and Statistical Manual of Mental Disorders – Fourth Edition (DSM-IV) [61] defined major depressive episode assessed using the Structured Clinical Interview for DSM (SCID) research version [62].

•Hamilton Depression Rating Scale -17 item (HDRS17) [63] score of ≥ 18 at week -2 and 0 (see below).

•Massachusetts General Hospital Treatment Resistant Depression (MGH-TRD) staging score of 2-10 as a measure of treatment refractoriness [64]. This cut off is based on MGH-TRD scores seen in primary and secondary care, but short of tertiary care, in the UK NHS [65].

•Single agent or combination antidepressant treatment which includes a serotonergic drug (a selective serotonin reuptake inhibitor, a tertiary amine tricyclic, venlafaxine, duloxetine or mirtazapine). At the point of randomisation, patients must have been on their current antidepressant medication, at the current dose, for a minimum of four weeks.

•Aged between 18-65. An upper age limit is included to reduce rates of physical health comorbidities that could complicate decisions around the safety of prescription of a drug (metyrapone) that may lower cortisol levels.

### Exclusion criteria

•Other DSM-IV axis I diagnosis, other than an anxiety disorder considered to be secondary to a primary diagnosis of depression, confirmed using SCID.

•Physical co-morbidity that would make metyrapone inappropriate, including untreated hypothyroidism, disorders of steroid production, cardiac failure, angina, myocardial infarction in the last 3 years and renal failure.

•Pregnancy or breastfeeding.

•Use of medication that would interfere with metyrapone.

•Dependence on alcohol or other drug(s) in the past 12 months, and/or current harmful use of such substances (defined as meeting SCID criteria for harmful use or dependence).

•Recent participation in a research study that could interfere with results.

### Recruitment

Patient identification will occur across two hubs of the UK NIHR Mental Health Research Network - the North East with centres in Newcastle and Teesside and the North West with Manchester as the centre - and in the West Yorkshire Comprehensive Local Research Network – with centres in Leeds and Bradford. Potential participants will be identified through routine clinic appointments at study sites and Participant Identification Centres that will include Primary Care and Community Mental Health Teams.

### Intervention

Participants will continue their existing antidepressant regime. Randomisation will be in a 1:1 ratio of metyrapone to matched placebo using permuted block randomisation, stratified by inclusion in mechanistic studies sub-sample, level of care setting (i.e. primary or secondary care) and by centre. A randomisation code will be computer generated by the Newcastle Clinical Trials Unit and coded packs of the study drug and matched placebo will be produced, according to the randomisation schedule. All medication will be supplied from a single central pharmacy with all participants and study staff blinded to treatment allocation. To ensure blinding the metyrapone will be over-encapsulated and the placebo will be visually identical. Participants in the treatment arm will receive study drug (metyrapone 500 mg or placebo) twice daily, prescribed in the morning and at noon, for 21 days. Adherence to medication will be assessed using measures of 11-deoxycortisol as described below under “HPA axis Assessment”.

Apart from treatment with the experimental intervention, all other treatments will remain under the control of the patient’s normal treating psychiatrist and/or general practitioner. However, the patients’ clinicians will be encouraged to avoid changes to medications between enrolment (week -2) and the primary outcome time point (week +5) unless there is a compelling clinical reason to alter treatment. All current medication will be recorded at all follow up time points.

### Objectives

#### Primary clinical objective

•The primary objective is to determine whether metyrapone (500 mg twice a day) for 21 days is efficacious in augmenting conventional serotonergic antidepressants in TRD in a UK NHS primary and secondary care setting. This to be assessed by Montgomery-Åsberg Depression Rating Scale (MADRS) [66] scores two weeks post treatment (week +5 from randomisation), comparing patients treated with metyrapone to those treated with placebo.

#### Secondary clinical objectives

•To determine the clinical effect size at two weeks post-completion of treatment of a three-week course of metyrapone (vs placebo) augmentation of antidepressants in depressed patients who have failed to respond to at least two courses of antidepressants, in primary care and psychiatric outpatient clinics in the UK.

•To assess whether the response is sustained for up to 21 weeks post cessation of metyrapone.

•To assess whether metyrapone augmentation improves patients’ quality of life using the self-completed EuroQol EQ-5D instrument (http://www.euroqol.org/).

•To assess the tolerability and safety of metyrapone augmentation in a large sample taken from a representative population of psychiatric outpatients and primary care patients with TRD.

#### Mechanistic objectives related to the full RCT sample

•To assess whether metyrapone changes patients’ HPA axis function.

•To assess whether changes in HPA axis function with metyrapone persist after stopping metyrapone.

•To assess whether the change in HPA axis function correlates with clinical response.

•To assess whether baseline HPA axis function predicts clinical response.

### Study schedule

All assessments will be undertaken by trained research personnel under the supervision of the clinically trained principle investigators (INF, RHMW, SW, IMA, AOH, HCRG, PMH, TH, AJL).

#### Screening visit (week -2)

Written informed consent will be obtained and study eligibility determined. Height, weight and safety measurements (see Table 2 for details) will be recorded. Baseline blood tests including urea and electrolytes, cortisol, thyroid function test, liver function tests, full blood count and β-human chorionic gonadotropin (if indicated) will be taken. Background factors, personality, and childhood adversity and life events will be assessed between recruitment and randomisation, using the NewMood background questionnaire [67] given to patients to complete and return at the next visit. This questionnaire includes the Big Five Inventory 44 personality questionnaire [68], a negative life events questionnaire adapted from the list of Life Threatening Experiences [69], the Social Circumstances Questionnaire, which is an adaptation of the Social Support Questionnaire [70] as used in the NewMood study [67], the Childhood Trauma Questionnaire [71] and the Ruminative Responses Scale [72]. Depression severity will be determined using the HDRS17, which will be rated using the GRID-HAMD for improved reliability [73], and the MADRS [66].

**Table 2:** Schedule of assessments

	**Enrolment**	**Randomisation**	**Follow up**							
**Time point**	**Week-2**	**Week 0**	**Week 1**	**Week 2**	**Week 3**	**Week 4**	**Week 5**	**Week 8**	**Week 16**	**Week 24**
Assessment of eligibility	√									
Informed Consent	√									
Assessment of baseline characteristics – NewMood questionnaire*	√									
Experimental Intervention										
Assessment of depression severity – HDRS17^1^	√	√								
Assessment of Clinical symptoms – MADRS^2^, CAS^3^, BDI^4^,STAI^5^, YMRS^6^		√			√		√	√	√	√
Assessment of Quality of Life – EQ-5D		√			√		√	√	√	√
Assessment of side effects – TSES^7^		√			√		√	√		
Assessment of side effects and adverse events – self report		√	√	√	√	√	√	√	√	√
Suicide risk assessment	√	√	√		√		√	√	√	√
Pregnancy Test if indicated	√	√	√	√	√					
Assessment of concomitant medication	√	√	√	√	√	√	√	√	√	√
Measurement of HPA axis function (CAR plus 11 pm saliva sample)		√			√		√			
Physical observations**	√***		√				√			
Blood Tests – U&E’s, cortisol	√****		√				√			

* - See text under “Screening Visit” for description of NewMood questionnaire details.

** - Physical observations comprised sitting and standing pulse and blood, and pressure respiration rate.

*** - Screening physical observations also included height and weight.

**** – Screening blood tests also including thyroid function tests, liver function tests and full blood count.

^1^*HDRS17* Hamilton Depression Rating Scale – 17 item.

^2^*MADRS* Montgomery-Asberg Depression Rating Scale.

^3^*CAS* Clinical Anxiety Scale.

^4^*BDI* Beck Depressive Inventory.

^5^*STAI* State Trait Anxiety Inventory.

^6^*YMRS* Young Mania Rating Scale.

^7^*TSES* Toronto Side Effects Scale.

#### Randomisation visit (week 0)

Subjects will excluded if their HDRS17 has dropped below 18 or if there has been any change in their current antidepressant medication (drug or dose). Otherwise study medication will be supplied to commence the following day.

#### Follow –up

Data will be collected at weeks +1, +2, +3 (end of active treatment period), +4, +5 (primary outcome time point), +8, +16 and +24 from the date medication was started (+/- 2 days). The week +2 and +4 visits can be completed by telephone. Details of the assessments at each time point are described in Table 2. Depression severity will be assessed using the MADRS administered by trained members of the research team at time +3, +5,+8, +16 and + 24 weeks. The primary outcome measure will be the change in MADRS from week 0 to +5 weeks. The MADRS has preferable psychometric properties and higher sensitivity to change than other depression rating scales [74] and was associated with the largest effect size in the Jahn study [33]. Additional secondary outcome measures of symptomatology (Clinical Anxiety Scale (CAS) [75], Beck Depressive Inventory (BDI) [76], State Trait Anxiety Inventory (STAI) [77] and Young Mania Rating Scale (YMRS) [78]) will be conducted at the same time points as described for the MADRS. Quality of life will be assessed using the self-completed EuroQol EQ-5D instrument (http://www.euroqol.org/) and tolerability using the Toronto Side Effects Scale (TSES) [79].

Metyrapone treatment potentially engenders hypocortisolaemia with manifestations including a risk of postural hypotension, hyperkalaemia and hypernatremia. Therefore, safety assessments will include serum cortisol measures at week +1 as well as measuring sitting and standing blood pressure and urea and electrolytes at weeks +1 and +5.

### HPA axis assessment

Cortisol levels to determine the cortisol awakening response (CAR) [80] will be obtained at the start of treatment (week 0) and then again at +3 and +5 weeks for all patients. This entails participants collecting 5 ml of saliva by passive drool [81] into a plastic collecting tube on wakening and then again at 15 minute intervals for a further hour. A total of five samples will be collected on each occasion either the day before or day after the planned study visit. Participants will also be asked to collect a saliva sample for cortisol assay at 11 pm the night before each of the three CAR assessments. In addition to collection of saliva samples, participants will complete a brief questionnaire relating to the nature and quality of sleep the night before the CAR assessment.

In addition to the saliva samples, serum samples will be taken at -2, +1 and +5 weeks for analysis of cortisol precursors and metabolites. Metyrapone administration has previously been shown to cause an increase in levels of ACTH, DHEA and 11-deoxycortisol together with an increase in the cortisone:cortisol ratio [33,82]. The increase in 11-deoxycortisol between weeks -2 and +1 will be used as a measure of adherence to medication since this has been shown to be highly sensitive to treatment with metyrapone [33,82].

### Sample size, power and effect size

The primary outcome measure is change in MADRS between weeks 0 and +5. The effect size for this measure in the Jahn study [33] was 0.63. The study has been powered around the more conservative moderate effect size of 0.5 which, assuming a post-intervention standard deviation of 12 points [33], corresponds to a six point difference on the MADRS. An achieved sample size of 85 per group is required to detect effect size of 0.5 with 90% power, assuming alpha = 0.05. Allowing for 10% attrition during the trial, the original aim was to randomise 95 per group; 190 in total. However, as described above the recruitment target has been modified accepting a power of 80%, requiring a sample size of 63 per group. Again allowing for 10% attrition during the trial, we aim to randomise 70 per group; 140 in total.

### Statistical analysis

A full Study Analysis Protocol will be drawn up prior to completion of the study and breaking of the study blind.

There is no planned interim analysis and no “stopping rules” for the study as a whole. Individual patients will be withdrawn from the study medication if it appears that to continue would be deleterious for their mental health or safety. This can be determined by the patient, the treating clinician and/or the research team and will be supported by the use of the MADRS particularly if there is an increase in the score for question 10 “suicidal thoughts”, or a lack of improvement in total MADRS score.

The primary outcome will be an intention to treat analysis of variance of the MADRS scores of the two treatment groups (metyrapone and placebo) at +5 weeks covarying for baseline MADRS. The persistence of change in the MADRS score will then be assessed using repeated measures analysis of variance utilising data from all time points. The change and persistence of the change in other clinical and quality of life measures will be examined using the same methods. Additional secondary outcomes will include rates of response (defined as a 50% or greater reduction in MADRS score) and remission (defined as MADRS ≤ 10) with metyrapone versus placebo.

With regard to the mechanistic objectives, the study will examine whether treatment with metyrapone leads to a change in HPA axis function (assessed by examining the CAR and 11 pm cortisol measures) and whether changes in HPA axis function, compared to baseline, are seen two weeks post treatment. Both baseline HPA axis function and change in function with treatment will be assessed to see if they predict clinical response to metyrapone defined by the change in MADRS between week 0 and week +5, in an exploratory analysis.

### Status of the study

The study was registered on 21/12/2009 (ISRCTN45338259) under the public title “Antiglucocorticoid augmentation of antiDepressants in Depression: the ADD study”. Clinical Trial Authorisation was given by the Medicines and Healthcare products Regulatory Agency (MHRA: EudraCT: 2009-015165-31). Ethical approval was granted by the Sunderland Local Research Ethics Committee (REC Ref No. 10/H0904/9) on 22/04/2010. Recruitment commenced in February 2011.

## Discussion

The ADD Study is the largest RCT of antiglucocorticoid treatment of Major Depression in the UK and one of the largest worldwide. In addition it is novel in its length of follow up and in its inclusion of a range of mechanistic studies exploring how such treatments might work. Compared to many previous studies, including a relatively large proof of concept study of metyrapone augmentation of conventional antidepressants [33], the ADD study is recruiting from a broad population of patients including those from both primary and secondary care. Inclusion criteria have intentionally been kept broad and exclusion criteria to a minimum in order to explore the efficacy of metyrapone treatment in as real world a setting as possible. This means, for example, patients with significant suicidal ideation, who are often excluded from RCTs in depression, will be included in the ADD Study.

The patients included in the ADD study will be extremely well characterised at base line in relation to a number of factors know to relate to TRD as well as influencing HPA axis function. For example personality traits are associated with refractoriness to treatment in patients with depression [83] and the ADD study will characterise personality using the Big Five Inventory 44 personality questionnaire [68]. Further, given the impact of life events and childhood trauma on HPA axis function and potential vulnerability to depression [84,85], these factors will also be well characterised using the negative life events questionnaire [69], the Social Circumstances Questionnaire [70] and the Childhood Trauma Questionnaire [71].

The dosing schedule for metyrapone that has been selected for the ADD Study is 500 mg twice a day administered in the morning and at noon. The total daily dose matches that given in previous studies [33]. The rationale for the timing of administration is to coincide with the portion of the day associate with the highest cortisol concentrations. While the ADD Study is not including direct measures of metyrapone plasma concentrations in patients, measurement of plasma 11-deoxycortisol after 1 week’s treatment will serve as a sensitive assessment of both compliance and pharmacodynamics effect of the treatment. Previous data shows that metyrapone administration is associated with highly significant increases in 11-deoxycortisol [33,82] due to its effect of blocking 11β-hydroxylase.

The degree of treatment refractoriness of patients will be carefully assessed using the MGH-TRD staging scale [64]. There is a lack of consensus as to which staging scale for refractory depression should be used in studies. We have chosen the MGH-TRD scale due to its ease of use especially taking into account scoring for dose optimisation and augmentation/combination treatment. We have chosen a minimum MGH-TRD score of 2 for inclusion. Use of a single antidepressant at an effective dose scores 1 point and hence this minimum represents a failure to respond to at least 2 antidepressants given usual UK practice in primary care of not augmenting or combining medications for depression until after this stage [1]. Beyond this point in the treatment algorithm for individual patients there is great divergence in practice with patients being referred to secondary care at different stages by individual clinicians. The maximum MGH-TRD score for inclusion has been set as 10. In practice this means 5-6 trials of different antidepressants allowing for dose optimisation and augmentation/combination strategies used for some of these trials. Use of electroconvulsive therapy scores 3 points in the MGH-TRD scale. As such this in itself is not an exclusion criterion in the ADD Study. However since most patients receiving ECT will have also had at least a couple of antidepressants, often with dose optimisation and augmentation, in practice few patients treated with ECT have a score under the maximum cut off of 10.

## Abbreviations

5-HT: 5-hydroxytrypatamine; ACTH: Adrenocorticotrophic hormone; ADD: Antiglucocorticoid augmentation of anti-depressants in depression; ANT: Attentional Network Test; BDI: Beck Depressive Inventory; BDNF: Brain derived neurotropic factor; CAR: Cortisol awakening response; CAS: Clinical Anxiety Scale; CRF: Corticotrophin releasing factor; DSM-IV: Diagnostic and Statistical Manual of Mental Disorders – Fourth Edition; ECMT: Emotional Categorisation and Memory Test; EEG: Electroencephalograpy; EEMET: Emotional episodic memory encoding task; ESMT: Emotional source memory task; FEERT: Facial Emotional Expression Recognition Test; FEPT: Facial emotional processing task; fMRI: Functional magnetic resonance imaging; HDRS17: Hamilton Depression Rating Scale – 17 items; HPA: Hypothalamic-pituitary-adrenal; LDAEPs: Loudness dependency of auditory evoked potentials; LTP: Long-term potentiation; MADRS: Montgomery-Asberg Depression Rating Scale; MGH-TRD: Massachusetts General Hospital Treatment Refractory Depression [staging score]; NICE: National Institute for Health and Clinical Excellence; NHS: National Health Service; OLM: Object-Location Memory; RCT: Randomised controlled trial; SCID: Structured Clinical Interview for DSM; SSRI: Selective serotonin reuptake inhibitor; STAI: State Trait Anxiety Inventory; SWM: Spatial working memory; TRD: Treatment refractory depression; TSES: Toronto Side Effects Scale; VTL: Verbal Learning Test; YMRS: Young Mania Rating Scale

## Competing interests

The authors declare that they have no competing interests.

## Authors’ contributions

The study was designed by INF, RHMW, SW, PG, IMA, EMcC NS. Funding was obtained by INF, RHMW, SW, IMA, AOH, EMcC, HCRG, PMH, TH, JW, FHW, AJL, INS. The manuscript was drafted by ES and RHMW. All authors reviewed, revised and approved the final version of the manuscript.

## Pre-publication history

The pre-publication history for this paper can be accessed here:

http://www.biomedcentral.com/1471-244X/13/205/prepub
